# High TNF-α and/or p38MAPK expression predicts a favourable prognosis in patients with T1N0M0 hepatocellular carcinoma: An immunohistochemical study

**DOI:** 10.3892/ol.2019.10781

**Published:** 2019-08-23

**Authors:** Mao Zhang, Jie Hu, Haoran Li, Shun Zhang, Weiyu Hu, Liqun Wu, Bing Han

Oncol Lett 17: 4948-4956, 2019; DOI: 10.3892/ol.2019.10193

Subsequently to the publication of this article, the authors have realized that the article contained several errors in need of correction. Firstly, the penultimate sentence in the “*IHC evaluation”* subsection of the Materials and methods on p. 4949–4950 that reads as “Patients with high TNF-α and low p38MAPK expression and patients with low TNF-α and high p38MAPK expression…” should have read as follows (changes highlighted in bold): “Patients with **high TNF-α expression alone** and patients with **high p38MAPK expression alone** were included in the TNF-α high-expression group and the p38MAPK high-expression group, respectively.”

Secondly, Fig. 2E-H in Fig. 2 should have been presented consistently with Fig. 3E-H in view of the fact that the high expression of TNF-α alone or the high expression of p38MAPK alone was compared with low expression of both TNF-α and p38MAPK. The revised version of Fig. 2, containing the correct presentation of Fig. 2E-H, is shown opposite. Note that the figure legend has not been altered as a consequence of these changes.

Finally, the labels written as “high and low” in Fig. 3E and F should have been written as “**TNF-α high**”, and the labels written as “low and high” in Fig. 3G and H should have been written as “**p38MAPK high**”, to make them tally with the description of the results in the article and be consistent with the figure legend. The corrected version of Fig. 3 is shown on the third page of this corrigendum. No modification of the description of the results relating to Figs. 2 and 3 has occurred as a consequence of these changes.

Note that none of these corrections affect the main conclusions reported in the paper. Nevertheless, the authors apologize to the readership of the Journal for any inconvenience caused.

## Figures and Tables

**Figure 2.**
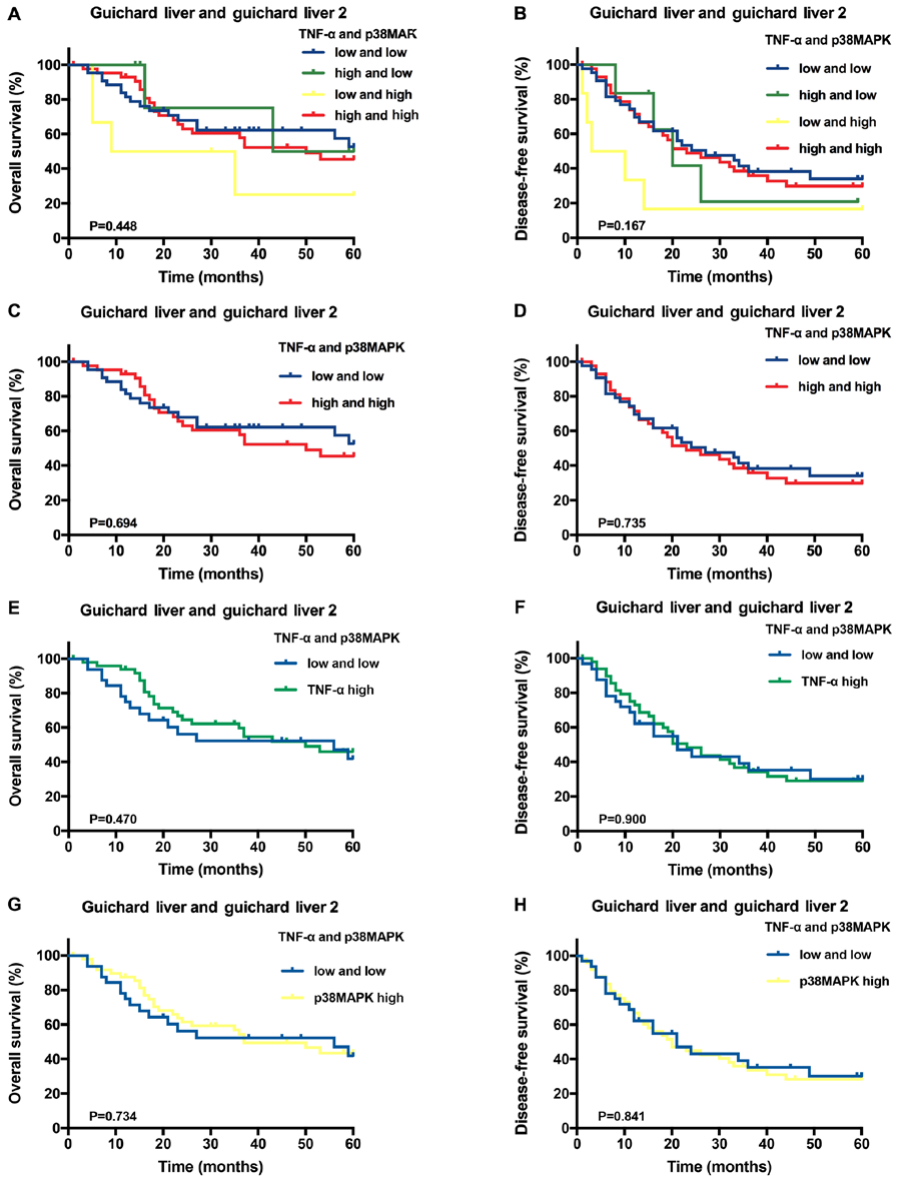
Predictive value of TNF-α and/or p38MAPK expression in the Oncomine™ database hepatocellular carcinoma samples. Kaplan-Meier overall survival and disease-free survival analysis of the different samples stratified according to the specific expression category: (A and B) both high vs. all others, (C and D) both high vs. both low, (E and F) TNF-α high vs. both low, (G and H) p38MAPK high vs. both low. TNF-α, tumour necrosis factor-α; p38MAPK, p38 mitogen-activated protein kinases.

**Figure 3.**
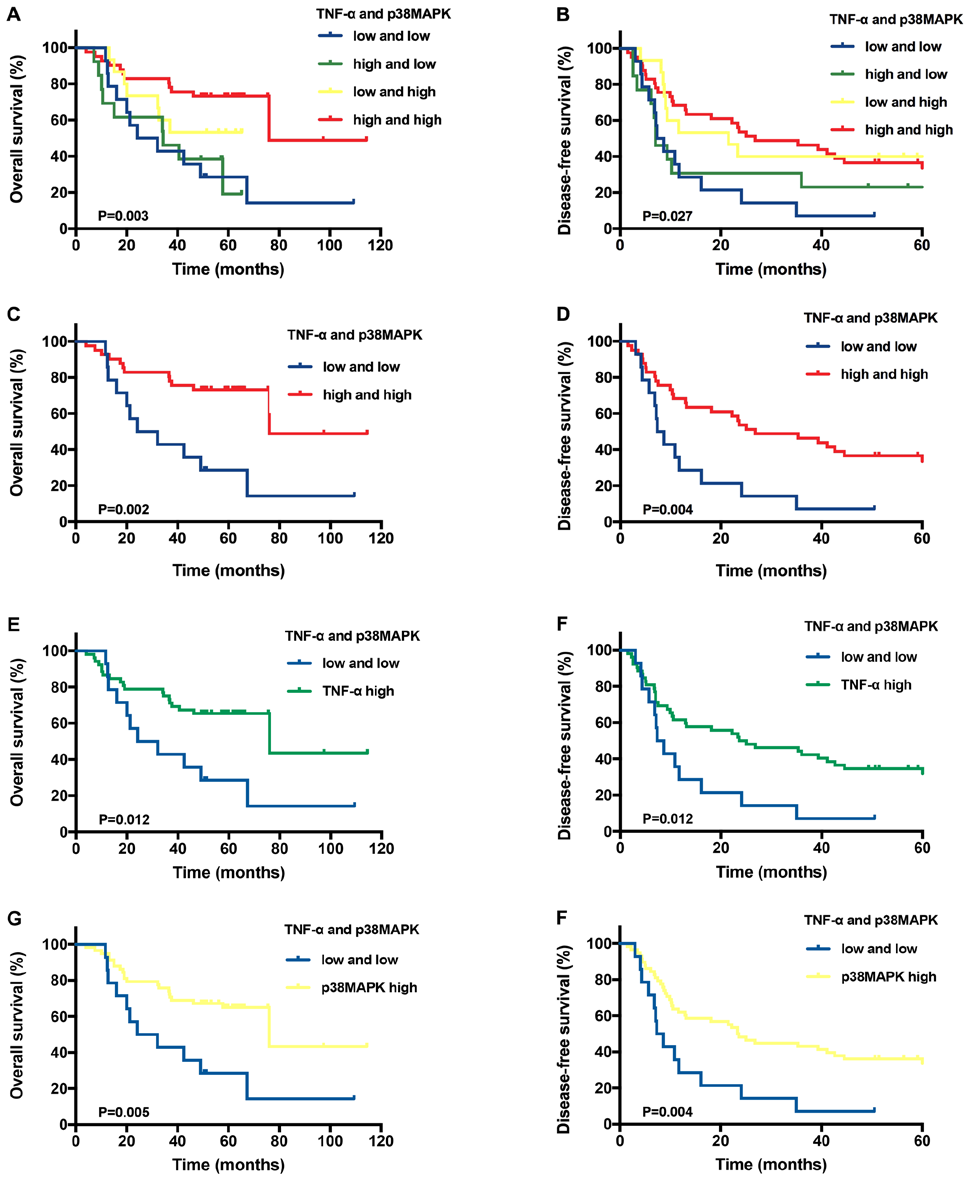
Predictive value of TNF-α and/or p38MAPK expression in patients with T1N0M0 hepatocellular carcinoma. Kaplan-Meier overall survival and disease-free survival analysis of the different tissues stratified according to the specific expression category: (A and B) both high vs. all others, (C and D) both high vs. both low, (E and F) TNF-α high vs. both low, (G and H) p38MAPK high vs. both low. TNF-α, tumour necrosis factor-α; p38MAPK, p38 mitogen-activated protein kinase.

